# In-Person and Online Sexual Harassment in the Workplace Against Women in Europe: A Structural Multilevel Analysis and the Aggravated Risk of the Young Cohort

**DOI:** 10.3390/bs16081309

**Published:** 2026-08-01

**Authors:** Cristina Cuenca-Piqueras, María José González-Moreno, Juan S. Fernández-Prados

**Affiliations:** Department of Sociology, Research Center for the Study of Migration and Intercultural Relations (CEMyRI), University of Almeria, 04120 Almeria, Spain; ccp693@ual.es (C.C.-P.); mgm302@ual.es (M.J.G.-M.)

**Keywords:** workplace sexual harassment, online harassment, digital gender-based violence, multilevel analysis, young cohort, prevention, structural risk

## Abstract

Workplace sexual harassment is a persistent human rights and public-health problem. This study analyzes in-person, online, and hybrid victimization using microdata from the EU-GBV survey (2021) across 17 countries, applying multilevel logistic models. The official indicator identifies that 31.6% of women have experienced harassment. In-person harassment is prevalent, whereas online victimization disproportionately affects the young cohort (18–34 years). Significant between-country variation is observed, indicating the influence of the national context in shaping risk. Among women under 35, urban environment and country-of-origin dynamics are relevant exposure factors. The findings show high recurrence (57.8%), low formal reporting (1.7%), and insufficient organizational preparedness (23.4% with training available). Digitalization expands the repertoire of violence without displacing its structural foundations. We conclude that there is a need to implement integrated prevention and intervention systems that address hybrid risk through a gender-sensitive perspective and a multivariable multilevel analysis attentive to intersecting axes, with models centered on main effects and the young cohort treated as an analytical stratification.

## 1. Introduction

Violence against women remains a major human-rights, public-health, and social-inequality problem across Europe. Its comparative measurement is methodologically difficult because police and administrative records capture only part of the phenomenon, while institutional differences between countries limit comparability. Large European survey studies have helped to correct this problem by making visible the extent of sexual harassment and other forms of gender-based violence beyond the criminal sphere (Goodey, 2017; Latcheva, 2017; Eurostat, 2022; FRA et al., 2024). Recently, Directive (EU) 2024/1385 (European Union, 2024) has strengthened the regulatory framework for combating these forms of violence within the European Union.

Within this broader field, sexual harassment requires conceptual precision. It must be situated within gender-based violence, but it should not be reduced to sexual violence in the strict sense. Recent reviews distinguish between legal-normative, socio-cultural, and organizational perspectives, and note that empirical research often uses partially overlapping definitions (Cuenca-Piqueras et al., 2023). At the European level, sexual harassment has long been identified as a widespread but often invisibilized form of gender-based violence, especially in contexts marked by power inequalities and normalized institutional routines (Latcheva, 2017). Workplace harassment (International Labour Organization, 2019) constitutes a violation of personal integrity and a critical psychosocial risk. Within the legal-normative perspective, sexual harassment is defined and operationalized strictly through codified statutory frameworks and institutional regulations established by administrative or legislative bodies, such as Directive (EU) 2024/1385; this perspective coexists with the socio-cultural and organizational approaches and, in empirical research, partially overlaps with them.

The recent literature suggests that workplace sexual harassment is best understood through at least three complementary approaches (Fitzgerald & Cortina, 2018; Cuenca-Piqueras et al., 2020). First, socio-cultural perspectives interpret harassment as a mechanism for reproducing gender, status, and inequality hierarchies. Second, organizational approaches explain it in relation to power structures, climates of permissiveness, and weak accountability mechanisms within workplaces (Cortina & Areguin, 2021). Third, multidimensional approaches hold that harassment is not a single act, but a repertoire of behaviors encompassing gender hostility, unwanted sexual attention, sexual coercion, and, increasingly, forms mediated by digital technologies (Ahuja & Padhy, 2021; Gómez-González et al., 2023; Flynn et al., 2024). To avoid conceptual conflation, we distinguish behavioral multidimensionality—Fitzgerald’s dimensions of gender hostility, unwanted sexual attention, and sexual coercion—from the intersectional approach, which examines how socio-demographic positions such as age and country of origin jointly shape differential vulnerability (Cuenca-Piqueras et al., 2023). Once these conceptual approaches are established, the question of their measurement follows: survey instruments translate these behavioral repertoires into operational indicators.

At the instrumental level, the measurement of workplace sexual harassment in Spanish-speaking contexts has gained methodological robustness thanks to recent cross-cultural adaptations. The Spanish-language adaptation of Fitzgerald’s Sexual Experiences Questionnaire-Workplace (SEQ-W) confirms that, when exploratory factor analyses are applied to samples of working women, the classic three-dimensional structure tends to collapse into a two-factor model composed of the dimensions of “Sexual Coercion” and “Unwanted Sexual Attention” (Soler-Sánchez et al., 2025). This psychometric phenomenon is attributed to a pronounced floor effect in general-population samples, where explicit conduct involving blackmail or direct coercion shows extremely low frequency and reduced variability compared with the subtle or hostile expressions of unwanted sexual attention (Soler-Sánchez et al., 2025). Consequently, the analytical distinction between in-person, virtual, and hybrid modalities proposed in this study engages not only with the typology of the conduct, but also with the sensitivity of the adapted instruments to capture the continuum of violence in organizational settings with different cultural matrices.

From a theoretical standpoint, we propose that the construct of hybrid harassment requires a profound reconceptualization that transcends the mere addition of isolated violent episodes, conceptually configuring it as a sociological category capable of capturing a unified continuum of violence reconfigured by the geography of digital work, the implementation of telework, and hybrid environments. Under this proposal, the digital transition in human-resource management and the widespread deployment of corporate and semi-private channels—such as instant-messaging tools, collaborative platforms, and asynchronous e-mail flows—would have dissolved the traditional spatiotemporal boundaries of the factory and office organization, institutionalizing dynamics of “spacelessness” and “timelessness.”

This deterritorialization of institutional boundaries drives a diffuse algorithmic subordination, forcing workers into a state of mandatory, omnichannel availability (Almeida et al., 2025; Bastante Velázquez, 2026). Such a scenario is associated with an increase in emerging psychosocial risks linked to information technologies, such as compulsory hyperconnection, technostress, and chronic technofatigue, transforming the assets of flexibility into vectors of imposition and psychophysical overload.

As the studies by Almeida et al. (2025) on working-time control demands in telework and by Bastante Velázquez (2026) on the intensification of socio-digital risk show, the demand for immediate responses distorts the exercise of managerial power and opens diffuse normative margins that foster the manifestation of moral and sexual harassment through corporate media. In our theoretical framework, we argue that aggression dynamics operate through interactive flows of online-to-offline and offline-to-online transition, in which digital tools do not act neutrally but rather as effective mechanisms of omnipresent monitoring, selective social exclusion, and chronic environmental hostility (Javed et al., 2023; Cuenca-Piqueras et al., 2020). This transmedia persistence of gender hostility is associated with an erosion of women workers’ space for privacy and mental health, inducing chronic states of anxiety and depression and a pronounced emotional exhaustion, or burnout, that drastically diminish their professional motivation (Almeida et al., 2025; Hitlan, 2026). The use of 2025–2026 references is deliberate: these contemporary frameworks provide the analytical tools to interpret the long-term structural consequences of telework and algorithmic management that shape the channels through which the dynamics captured by the 2021 survey continue to manifest.

From a sociological conflict perspective, this digital displacement hyperconcentrates violence in time and in the victim’s perceived space, drastically reducing her “space for action” and her capacity for resistance within the organization. This phenomenon of unprotectedness and defenselessness is particularly frequent among the youngest and most precarious cohorts of the labor market, whose positional vulnerability and contractual dependence limit the possibilities of activating effective reporting resources (Gómez-González et al., 2023; Hitlan, 2026; Javed et al., 2023). In line with what Javed et al. (2023) argue in their analysis of cyberbullying in remote employment, the overlapping of corporate and informal channels transforms the virtual environment into an arena of structural unprotectedness, where pre-existing power asymmetries become more sophisticated rather than neutralized. Consequently, it is pertinent to formulate an epistemological critique of the analytical bias of prior literature, which has tended to compartmentalize and study in a dichotomous and segregated manner the expressions of virtual (online) and physical (offline) violence, as if they constituted watertight and disconnected scenarios (European Institute for Gender Equality [EIGE], 2017; Cuenca-Piqueras et al., 2020). This theoretical oversimplification ignores that digitalization neither replaces nor dilutes the classic structures of gender domination and organizational hierarchies, but rather expands, diversifies, and intensifies the repertoire of hostile, environmental, and coercive behaviors directed against women (Cuenca-Piqueras et al., 2020). As such, a central focus of this study is the young cohort of women workers aged 18–34, who are in the stage of early labour market entry and occupational transition.

A comprehensive 360-degree approach requires mapping an expanded risk continuum that recognizes the effects of polyvictimization and the systemic accumulation of direct and indirect harassment tactics (Hitlan, 2026). The landmark transnational study by Cuenca-Piqueras et al. (2020) shows that generational factors and the national socio-institutional context intersect decisively and significantly in defining the prevalence and differential nature of face-to-face versus virtual harassment. Likewise, as Hitlan’s (2026) model demonstrates the co-occurrence of multiple vectors of organizational mistreatment— including the additive impact of direct harassment and bystander harassment—irreversibly undermines staff’s psychosocial coping resources, raising anxiety and precipitating work and job withdrawal behaviors. Isolation and unstructured rotating shifts compound these risks, maximizing worker vulnerability due to the absence of formalized support networks (Jonsdottir et al., 2022). This integrated, 360-degree understanding of the risk continuum underscores why the preventive dimension is decisive.

This preventive dimension is crucial because evidence and response infrastructures remain insufficiently developed in many work settings. Gómez-González et al. (2023) argue that workplace prevention requires not only legal definitions, but also risk-assessment tools that capture harassment and barriers to reporting. From a policy standpoint, Mergaert et al. (2023) propose the 7P framework—prevalence, prevention, protection, prosecution/sanction, provision of services, partnerships, and policies—as a way to integrate gender-based violence into a broader institutional architecture. This preventive dimension is presented as the institutional counterpart to the behavioral dimensions of harassment and provides the baseline for the study’s fourth objective.

The present study investigates workplace sexual harassment against women using the 2021 wave of the EU-GBV across 17 European countries. The objectives are fourfold: (1) to estimate the prevalence of in-person, online, and hybrid workplace sexual harassment across age groups; (2) to conduct a coverage and construct-decomposition analysis of the derived indicators against the survey’s official summary; (3) to assess, through multilevel models, the extent to which in-person and online harassment vary across countries once individual covariates are controlled for; and (4) to examine repetition, disclosure/reporting, and organizational preparedness as key dimensions for prevention, education, and intervention.

This study addresses two gaps in the literature: first, it transcends dichotomous frameworks by evaluating the physical–digital continuum through derived indicators from a harmonized transnational survey; second, it pairs multilevel risk assessment with an analysis of organizational preventive infrastructures. The cross-national component is not merely exploratory: we expect between-country variation to remain significant after controlling for individual characteristics, reflecting distinct macro-institutional and gender-equality regimes across the 17 countries.

## 2. Materials and Methods

### 2.1. Study Design and Data Source

This study performs a secondary analysis of microdata from the 2021 wave of the EU survey on gender-based violence against women and other forms of interpersonal violence (EU-GBV). Eurostat, the European Union Agency for Fundamental Rights (FRA), and the European Institute for Gender Equality (EIGE) jointly developed the EU-GBV, with national fieldwork coordinated across multiple Member States. It provides harmonized information on lifetime violence and violence in the last 12 months, including specific modules on sexual harassment in different contexts. Initial results were disseminated by Eurostat (2022, 2024) and by FRA et al. (2024).

The analytical file used in this study is a harmonized working dataset containing 94,392 women aged 18 to 74 residing in 17 European countries: Austria, Belgium, Bulgaria, Croatia, Denmark, Slovakia, Slovenia, Spain, Estonia, Finland, France, Greece, Latvia, Lithuania, Malta, Poland, and Portugal. Because the current dataset covers 17 countries rather than the full EU-27, the descriptive and multilevel estimates apply strictly to this specific empirical scope.

### 2.2. Population, Sample, and Technical Specifications

The target population was women with work experience, given that the items on sexual harassment at work refer to the entire working life. Filtering the original 94,392 observations yielded an analytical sample of 87,979 women with work experience and valid data. Within the young cohort (18–34 years), the initial 21,557 women were narrowed down to 18,880 with work experience. The 17 countries correspond to the complete set of Member States available under the Eurostat research contract for the 2021 wave; ‘work experience’ denotes any lifetime paid employment (full- or part-time), of which 74.2% were currently employed and 25.8% had past experience at the time of collection. In the multilevel models, listwise deletion of cases with incomplete covariates further reduces these figures to 84,950 (general) and 18,162 (young) before the exclusion of cases lacking a valid degree-of-urbanization classification (chiefly the Estonian subsample; see Section 4.6), yielding the final estimation samples of 81,760 and 17,280, respectively.

The methodological decision to isolate and explore autonomously the segment of women under 35 with work experience responds to theoretical and empirical criteria firmly supported by contemporary sociological literature. From the life-course perspective, the threshold of 35 years delimits the critical period of early labor-market entry and occupational transition, a stage structurally marked by greater exposure to temporary contracts, lower levels of formal authority within organizations, and an accentuated positional vulnerability. Likewise, at the transnational epidemiological level, cohorts aged 18 to 34 persistently record the highest crude prevalence rates of victimization in hybrid professional environments, being particularly affected by the intersection between material labor precarity and the dynamics arising from digital socialization (Jonsdottir et al., 2022).

The technical characteristics of the working file are also relevant for interpretation. The data structure relies on two features: first, observations are nested within 17 countries to enable multilevel modeling; second, the analyses remain unweighted, despite the sampling and calibration weights included in the EU-GBV file. The aim is not to estimate population prevalences, but rather the associations between individual characteristics and victimization and the between-country variance, a purpose for which unweighted maximum-likelihood estimation is more appropriate. Consequently, the descriptive prevalences offered here are proportions of the analytical sample and are not directly comparable with Eurostat’s official weighted figures, which are used solely as a contextual reference. The study followed a secondary-analysis strategy focused on replicability, transparency, and prudence in the interpretation of derived constructs. This distinction aligns with the study’s first objective: the descriptive figures map sample prevalence within the analytical sample, whereas the multilevel models use unweighted data to estimate associations and between-country variance rather than population counts.

### 2.3. Measures and Operationalization

The instrument includes a battery of questions on workplace sexual harassment asking whether respondents had experienced unwanted behaviors at work. The instrument comprises ten items on unwanted behaviors; their exact official wording and the corresponding interviewer instructions are documented in the survey’s methodological manual (Eurostat, 2021, Manuals and Guidelines KS-GQ-21-009, p. 88). These items assess lifetime prevalence—whether respondents had ever experienced the behaviors during their working life. The entire module is prefaced by an institutional filter binding all items to the respondent’s work environment (colleagues, supervisors, clients, or third parties), thereby excluding generic non-work digital exposure. Throughout, ‘online’ and ‘digital’ are used interchangeably to denote technology-mediated victimization.

To distinguish analytically between different modalities of workplace harassment, two derived indicators were constructed. The in-person indicator combines the six items describing direct interpersonal behaviors: inappropriate staring or leering; indecent sexual jokes or offensive remarks; inappropriate suggestions to go out on a date; inappropriate suggestions for any sexual activity; unsolicited physical contact; and threats of unpleasant consequences for refusing sexual proposals or advances. The online indicator comprises exclusively the two items whose official wording explicitly names a digital channel: inappropriate advances on social networking websites, and inappropriate sexually explicit emails or text messages. The item on exposure to sexually explicit images or videos was assigned to neither channel indicator, as its wording does not specify the channel of exposure and such exposure is frequently technology-mediated; the residual ‘other similar behaviour’ item was likewise excluded owing to its heterogeneity. Except for staring and unsolicited physical contact, which are intrinsically face-to-face behaviors, the official wording of the in-person items does not specify the channel of occurrence; their assignment to the in-person indicator follows a conservative rule under which only items explicitly naming a digital channel enter the online indicator. A hybrid indicator identified women who simultaneously presented the in-person and online indicators. It should be clarified that, given the cross-sectional nature of the EU-GBV survey, this hybrid indicator refers strictly to the co-occurrence of both modalities (simultaneous reporting of physical and digital victimization) and should not be interpreted as a temporal sequence or evolution of events. Because each item is answered independently on a dichotomous (Yes/No) scale, the derived hybrid indicator does not assume double counting: it identifies respondents who independently reported both an in-person and an online item.

The derived indicators were contrasted with the survey’s official summary of workplace sexual harassment. The coverage of the derived indicators relative to the survey’s official summary was checked before modeling, in order to delimit the extent to which the derived modalities could be used in the multilevel analysis without overinterpreting measurement artifacts. The official summary indicator is a binary composite triggered when a respondent reports at least one of the eight behaviors; the coverage check confirmed that the cases captured by the derived in-person and online sub-indicators were structurally nested within this composite.

The survey also provides work-specific indicators on perpetration, repetition, disclosure/reporting, and organizational preparedness. Perpetrators include coworkers, bosses or supervisors, clients, patients, students, or other people in the work environment. Repetition is measured by whether the same perpetrator engaged in harassing behavior more than once; recency, by whether the most recent episode occurred within the previous 12 months. Disclosure/reporting includes talking to someone close, to a coworker, to a manager or supervisor, to the police, or to another official body. Organizational preparedness is captured through the availability of training at work, the existence of a designated contact person, and knowledge of where to seek help.

### 2.4. Covariates

The multilevel models included age group, educational level, degree of urbanization, country of birth, and the ability to cope with an unexpected expense as a proxy for financial strain. Age was grouped into six bands (18–24, 25–34, 35–44, 45–54, 55–64, and 65–74). Educational level was coded into three broad categories (low, medium, high). The degree of urbanization distinguished between densely populated, intermediate, and sparsely populated areas. Country of birth was recoded into native-born versus foreign-born. The financial-strain variable distinguished between those who could and those who could not cope with an unexpected expense. Educational level follows ISCED 2011 (Low = ISCED 0–2; Medium = ISCED 3–4; High = ISCED 5–8) and the degree of urbanization follows DEGURBA (Cities = densely populated; Towns/Suburbs = intermediate; Rural = sparsely populated). The reference categories in the mixed-effects models are High education (ISCED 5–8), Rural areas, Native-born, and ‘can cope with an unexpected expense’.

### 2.5. Analytical Strategy

The analysis was carried out in four sequential stages. First, through descriptive exploration, age group mapped the prevalence of in-person, online, and hybrid harassment. Second, a descriptive analysis and country ranking of in-person and online harassment were likewise carried out. Third, multilevel logistic models were estimated for in-person and online harassment, allowing each country to have its own random intercept, both for the population as a whole and for the young-population subsample (18–34). Finally, key variables such as harassment repetition, reporting, and organizational preparedness were examined in order to deepen the discussion on prevention.

Generalized Linear Mixed Models (GLMM) provide the basis for the multilevel modeling. This statistical framework accounts for the clustering of individual observations within countries, separating personal characteristics from national-level variations. The methodological approach helps mitigate the potential WEIRD bias (Western, Educated, Industrialized, Rich, and Democratic) by isolating the contextual variance not explained by individual variables and providing more robust estimates of the fixed effects. The two outcome variables are binary indicators: the probability of lifetime in-person and of lifetime online workplace sexual harassment, respectively.

Regarding the interpretation of the results, a standard reparameterization of the target event was performed in the models estimated with the GENLINMIXED procedure in IBM SPSS Statistics version 30 (IBM Corp., Armonk, NY, USA). Because this procedure models the reference category by default, the event was redefined so that an odds ratio (OR) greater than 1 indicates a higher risk of victimization, facilitating the reading of risk factors.

## 3. Results

### 3.1. Descriptive Patterns of Workplace Sexual Harassment

The survey’s own official architecture already indicates that workplace sexual harassment is not a marginal phenomenon. The survey database identifies 29,839 women (31.6%) as victims of at least one form of sexual harassment at work over their working life. This figure is consistent with previous European literature underscoring the breadth of the problem, although it is not directly comparable with the EU-27 estimates disseminated in official reports because of the different country scope of the dataset used here. Table 1 refines this picture by separating in-person, online, and hybrid modalities. In-person harassment was the most prevalent form over the life course, declining from 48.2% among women aged 18–24 to 19.8% among those aged 65–74. Online harassment also showed a marked downward gradient, from 19.0% to 2.1% in the same age groups. Hybrid forms—women exposed to both in-person and online items—fell from 17.4% in the youngest to 1.6% in the oldest. The mean item counts reflected the same pattern: intensity was always higher among young women and decreased progressively with age. This pattern suggests that the virtual environment constitutes a relevant arena of victimization for the new generations of female employees. The 31.6% is computed over the full working dataset of 94,392 women; the modality-specific rates reported in Table 1 are proportions of the analytical sample (*N* = 87,979).

The lower lifetime prevalence reported by older cohorts, although counterintuitive for a lifetime measure, is a well-documented pattern in gender-based violence surveys: it reflects a cohort effect (older women entered labour markets with more normalized hostile behaviors), recall decay over a longer life course, and a higher subjective threshold for labelling behaviors as harassment relative to the more sensitized younger cohort.

The analysis of hybrid forms reveals a critical finding for understanding the phenomenon: digital victimization does not operate as an isolated modality, but rather as a vector of persistence and continuity of in-person violence. With an overall prevalence in which 88.6% of women who experience online harassment simultaneously report in-person incidents, the hypothesis of a ‘substitution’ of physical violence by virtual violence is ruled out. Because the survey does not link perpetrator identity across items, this pattern is interpreted as structural environmental continuity rather than as the trajectory of a single perpetrator.

This interconnectivity reaches critical levels in the young cohort (18–34 years), where the overlap of modalities affects more than 90% of online-harassment victims (91.5% in the 18–24 group and 90.5% in the 25–34 group). This figure not only denotes a cumulative vulnerability, but also underscores the existence of a ‘totalized’ work ecosystem for the youngest workers: an environment where the boundaries between physical presence and digital footprint are nonexistent for the perpetrator, who uses the ubiquity of digital channels to ensure the effectiveness of in-person coercion. For the new generations, harassment is not a dichotomous experience (in-person versus digital), but a continuous and reinforced experience, in which digital mediation acts as a mechanism of monitoring, surveillance, and re-victimization that transcends the physical space of the workplace.

### 3.2. Country-Level Patterns: Parallel Classification (General Population Versus Young Cohort)

The layout of this parallel double-classification matrix (see Table 2) allows a direct analytical reading of the structural continuities and generational ruptures that define workplace sexual harassment in the European Union. The first conclusive element emerges when comparing the aggregate totals in the last row: the young cohort experiences a systematic intensification of risk in both interactive-environmental arenas. On the in-person side, crude prevalence climbs from an already critical 32.5% in the general population to 45.6% among women under 35 (a net increase of 13.1 percentage points). This asymmetry sharpens in the digital dimension, where the rate recorded by young women workers (16.5%) almost doubles the all-ages continental baseline (8.5%), confirming that the virtual environment constitutes a preferential vector of aggression for the new generations of female employees. Hybrid harassment is not modeled as an independent dependent variable because its high overlap with online victimization (88.6% co-occurrence) would introduce redundant estimates and collinearity.

The left wing of the table reveals that the macro-national distribution of in-person harassment shows a notable hierarchical symmetry between the general population and the young subgroup. France and Finland co-lead both rankings of direct physical exposure, with rates exceeding 65% in the young cohorts, while Bulgaria and Latvia consistently close the last positions of the continental record. By contrast, the right wing of the matrix (online harassment) completely breaks the traditional geographic inertia, placing Slovakia, Estonia, and Slovenia at the top. This shift of the digital-risk focus toward the Eastern European axis is visible across all ages, but it is notable in the under-35 segment, where Slovakia records an online-harassment prevalence of 33.3%, an approximate 60% increase over its national general rate of 20.9%. Sociologically, this crossed pattern is consistent with the hypothesis that two independent macro-dynamic forces fuel the continuum of gender-based violence: on the one hand, the Nordic paradox of equality, which raises in-person self-reporting in Western democracies with high awareness. On the other hand, the unprotected affect of the international division of digital labor and the gig economy in Eastern Europe, which commodifies and virtualizes hostile work interactions, severely penalizing its youngest women workers. The statement that Bulgaria and Latvia occupy the lowest positions refers to aggregate all-ages general prevalence. The ‘Nordic paradox’ and ‘Eastern gig-economy’ readings are advanced strictly as post hoc theoretical propositions for future verification, not as causal findings of the models.

### 3.3. Multilevel Analysis of Online and In-Person Workplace Harassment (General Population)

The models incorporated the substantive variables as fixed effects (age, educational level, degree of urbanization, country of birth, and financial strain). The estimates proved robust to this specification, allowing a more precise estimation of the between-country variance.

In the general-population models (Table 3), age group is the strongest fixed effect, with young women showing markedly higher adjusted odds of online victimization than the oldest cohort; lower educational level is associated with lower reported odds of both in-person and online harassment, and residence in cities increases exposure relative to rural areas.

The multilevel model fitted for in-person harassment maintained a significant random intercept at the country level. As Table 3 shows, the model included 81,760 women in 17 countries, with a between-country variance of 0.637 (*p* = 0.005; adjusted ICC of 12.3%). It should be noted that these values were computed using the delta method. The lower prevalence of online harassment compresses its ICC on the probability scale, which explains why its ICC is smaller (6.8%) than that of the in-person model (12.3%), despite the online model presenting greater between-country variance (0.972 versus 0.637). age emerged as the strongest block, accompanied by education, urbanization, country of birth, and financial strain. All models achieved convergence under Newton–Raphson estimation with positive-definite Hessians.

### 3.4. Multilevel Analysis of Online and In-Person Workplace Harassment (Young Cohort, 18–34 Years)

Table 4 presents the estimates of the multilevel logistic models (GLMM) for in-person and online harassment in the cohort of women aged 18 to 34 (*N* = 17,280 and 17,212, respectively). The models were parameterized to estimate directly the probability of experiencing harassment (Y = 1), so that odds ratios (OR) greater than 1.00 indicate higher risk and those below 1.00 indicate lower risk, relative to each reference category. In this cohort, the degree of urbanization is the most robust correlate: residing in cities multiplies the risk of in-person harassment relative to rural areas (OR = 1.49). Low educational level is associated with lower reported in-person harassment (OR = 0.72), and financial strain shows no significant effect on in-person harassment (OR = 1.00), although it does raise online harassment (OR = 1.12). Between-country variance remains of a magnitude similar to that of the general population (σ^2^ country = 0.658 for in-person and 0.924 for online; *p* ≤ 0.006), although the ICC—dependent on prevalence through the delta method—is somewhat higher in this cohort, especially for online harassment (11.0% versus 6.8% in the general population).

### 3.5. Repetition, Disclosure/Reporting, and Organizational Preparedness

Workplace sexual harassment is not only prevalent but also frequently recurrent. Among respondents with valid information on repetition, 57.8% reported having experienced repeated sexual harassment at work by the same perpetrator (Table 5). Likewise, among cases with information on recency, 14.3% indicated that the most recent episode had occurred within the previous 12 months. These figures reveal that victimization in the work environment has a chronic and protracted character, and that a substantial minority of the sample experiences recent exposure.

Disclosure patterns point to a marked institutional asymmetry. Among victims in the last 12 months, talking to someone close (59.5%) or to a coworker (49.6%) was much more frequent than turning to a manager or supervisor (23.1%); formal reporting to the police (1.7%) or to another official body (1.2%) was exceptional. On the preventive side, only 23.4% of women workers reported having sexual-harassment training available at their workplace and 37.2% a designated contact person, whereas 66.7% stated they knew where to seek help. These organizational indicators are analyzed at the aggregate level because the module gauges company-wide structural resources rather than individual demographic exposure; sub-stratification by age would substantially reduce the contextual denominators.

## 4. Discussion

### 4.1. Main Findings: Continuum, Age Gradient, and Between-Country Variance

The present study examined workplace sexual harassment against women through an integrated framework combining gender power, organizational conditions, digital mediation, and transnational variation. The results clearly show that in-person harassment remains the dominant form of workplace victimization, whereas online harassment constitutes a distinct modality, less frequent but substantively important, concentrated with particular intensity among young women. Moreover, between-country variation persists once multiple covariates are adjusted for, suggesting that workplace sexual harassment cannot be explained solely by individual characteristics. In this regard, it should be noted that the hybrid-harassment category used in this analysis describes the statistical co-occurrence of reports in a cross-sectional survey, identifying situations of simultaneous reporting without presupposing a temporal or causal trajectory between the in-person and the virtual.

From a theoretical standpoint, the results support an interpretation in terms of a continuum and an accumulation of subtle and hostile tactics, rather than a modal-substitution model. In-person harassment remains the dominant substrate of workplace victimization, while digital forms are observed as an expansion of its repertoire. This trend is consistent with the understanding that technologies are associated with pre-existing inequalities, displacing interaction toward hybrid spaces where normative and evidentiary boundaries are more diffuse (Cuenca-Piqueras et al., 2020; Ahuja & Padhy, 2021; Flynn et al., 2024). Along these lines, the systematic review by Rogers et al. (2023) describes how technology-facilitated abuse (TFA) relates to the domains of the Duluth Power and Control Wheel, with key mechanisms of gender-based violence observed through corporate and semi-private digital channels. Using this conceptualization, the authors explain how the perpetrator’s perceived digital ‘omnipresence’ and ‘omniscience’—associated with the technological tools the worker uses—relate to a reduction in women workers’ ‘space for action’ and resistance. This state of chronic surveillance and forced connectivity is not only linked to the persistence of exposure to harassment, but is also associated with chronic states of anxiety and polyvictimization, with a convergence of physical and virtual threats observed in a unified experience of risk. This phenomenon suggests that digitalization is associated with a concentration of violence in the victim’s time and perceived space. Specifically, Rogers et al. (2023) map technology-facilitated abuse onto the domains of the Duluth Power and Control Wheel, where the perpetrator’s digital ‘omnipresence’ and ‘omniscience’—enabled by mandatory corporate tools—compress the survivor’s ‘space for action’ and sustain chronic exposure.

The age gradient deserves special attention. In the present data, online harassment is concentrated much more among young women than in-person harassment. This pattern is consistent with previous European evidence on greater digital exposure and greater vulnerability in early life stages, but it also suggests an articulation between labor position, digital socialization, and lower organizational power. Young women tend to be overrepresented in more precarious or dependent positions, and that imbalance appears to relate to situations in which control, availability, and interaction shift to digital and semi-private channels. Comparatively, the age gradient is markedly steeper for online than for in-person harassment, indicating that digital spaces are strongly generation-dependent arenas of risk.

The country-level variance revealed by the multilevel models is theoretically significant and calls for a cautious interpretation. The results confirm that both in-person and digital harassment maintain significant random intercepts, which indicates that the national context influences the risk of victimization beyond individual characteristics. Nonetheless, it is imperative to clarify a limitation of the scope of the present study: although the model structure confirms the statistical relevance of between-state variance, the current analysis does not include level-2 macro-social predictors nor does it empirically identify the specific national mechanisms. In this regard, we propose two explanatory hypotheses as theoretical propositions for future research: (1) the “Nordic paradox hypothesis,” which suggests that high levels of in-person reporting in countries with greater formal equality might relate to greater awareness and visibility of the phenomenon; and (2) the “Eastern gig-economy hypothesis,” which posits that the high digital prevalence in certain emerging economies might be linked to greater unprotectedness in the new virtual-service sectors. These interpretations, therefore, do not constitute causal findings of the model, but rather analytical axes for deepening the understanding of how regulatory frameworks and organizational cultures shape the map of harassment in Europe.

The East–West divergence, with digital-harassment rates of 33.3% in Slovakia and 29.8% in Estonia for young cohorts, reflects the transformation of the digital economy and the dissolution of work boundaries. The outsourcing of services and the absorption of female employment into telework and the gig economy (Marczak et al., 2026) are associated with the displacement of hostile behaviors toward virtual environments. Kocabaşoğlu et al. (2023) observe that 73.6% of the professional women surveyed reported sexual cyberharassment on corporate platforms and tools, mainly through dating requests and gender-based insults. This digital environment triggers psychological and physiological effects comparable to physical hostility—such as anxiety, diminished motivation, and job abandonment—while enabling Digital Intimate Partner Violence (DIPV) to manifest within organizational dynamics through corporate devices (Hearn et al., 2023). This phenomenon operates across two dimensions: “spacelessness,” where domestic violence penetrates professional channels like email and instant messaging (Fathimah et al., 2023), and “timelessness,” where the permanent availability demanded by algorithmic management blurs working hours and extends victimization risks beyond formal organizational control. Consequently, these shifts redefine power asymmetries (Herod, 2025) and introduce critical challenges for organizational ethics, information security, and employer liability. Ultimately, because the use of corporate infrastructure for DIPV compromises data integrity and expands risk across digital assets, organizations must urgently revise their legal compliance and liability protocols.

### 4.2. Risk and Vulnerability of the Young Cohort: Intersecting Axes

The submodel for the young cohort (*N* = 17,280) utilizes a multivariable multilevel analysis to evaluate intersecting risk axes, treating youth as an analytical stratification rather than a statistical interaction to transcend age as an isolated variable. Contractual instability during early career stages articulates with algorithmic-management systems to drive this intensified risk. Furthermore, positional vulnerability is closely tied to urbanization: women residing in cities face a substantially higher risk of in-person harassment than those in rural areas (OR = 1.49). This disparity reflects the concentration of precarious employment and service-sector roles that demand high third-party interaction within urban centers. In this setting, the framework of Gómez-González et al. (2023) on the intensification of socio-digital risk is essential, since the opacity of algorithmic control and the dissolution of professional boundaries exacerbate pre-existing power imbalances. Regarding country of birth—taking native-born women as the reference—risk is modestly higher among those born in another EU country (OR = 1.20), but significantly lower among those born outside the EU (OR = 0.84). This pattern qualifies the assumption of a homogeneous migrant vulnerability: the protective effect observed among those born outside the EU might reflect lower exposure to the work environments where hybrid harassment manifests with greater visibility, or else under-reporting phenomena linked to structural position. For its part, low educational level operates as a factor of lower reported harassment (OR = 0.72), and financial strain shows no significant effect on in-person harassment in this cohort (OR = 1.00). The lower reported odds among lower-educated women reflect a reporting and labelling bias rather than genuine protection—higher-educated women have greater resources to recognize and report hostility—while the non-significance of financial strain for in-person harassment indicates that the structural vulnerability of youth cuts across income levels. In line with Reuter et al. (2020), precarious employment, marked by schedule unpredictability and limited information about occupational risks, is associated with a higher prevalence of unwanted sexual attention, compounding this positional vulnerability.

### 4.3. Institutional Architecture of Prevention: From the 7P Framework to Operational Guidelines

Translating the structural findings into organizational guidelines requires the adoption of the analytical 7P framework (Prevalence, Prevention, Protection, Prosecution/Sanction, Provision of Services, Partnerships, and Policies) proposed by Mergaert et al. (2023), articulated with standards such as the Belgian guide (Federal Public Service Employment, Labour and Social Dialogue, 2020) and the Irish code (Irish Human Rights and Equality Commission, 2022). This preventive architecture must be comprehensive and transcend mere awareness-raising in order to integrate harassment into the psychosocial risk-management cycle (Federal Public Service Employment, Labour and Social Dialogue, 2020). The effectiveness of this management depends on a rigorous distinction between consolidated ‘good practices’—such as the standardized procedures of the Irish Human Rights and Equality Commission (2022)—and ‘promising practices,’ which require longitudinal validation given the risks of the digital environment (Mergaert et al., 2023).

In operational terms, the 7P framework requires the employer to implement participatory risk analyses to gauge Prevalence and Prevention, ensuring that training is universal and adapted to out-of-hours communication (Irish Human Rights and Equality Commission, 2022). Protection and Provision of Services must guarantee non-revictimization through multiple confidential points of entry.

Finally, institutional credibility is grounded in Prosecution/Sanction, social Partnerships, and the development of transmedia Policies. The latter must include reporting channels that allow digital evidence to be documented and periodic audits of communication systems to protect especially the young cohort from technology-mediated harassment.

### 4.4. Education and Capacity Building

The current findings also point to a significant educational deficit. If only a limited proportion of respondents report training available, then many organizations continue to rely on formal policy without integrating it into everyday learning. This is especially problematic in hybrid and remote contexts, where digital interaction can normalize ambiguous behaviors that require explicit clarification.

At the most basic level, organizations need universal education for the entire workforce: onboarding materials, periodic refresher modules, and examples that clarify what counts as in-person and online sexual harassment. Training must also be directed specifically at managers, supervisors, human resources, and contact persons, since procedural credibility depends on their ability to recognize signs, document facts, avoid secondary revictimization, and activate proportionate and safe response routes.

Recent intervention-oriented research provides useful design principles. Flynn et al. (2024) report that sector stakeholders repeatedly recommended that training on technology-facilitated sexual harassment at work be adapted to specific contexts, updated regularly, and integrate issues such as digital evidence, privacy, out-of-hours communication, and internal reporting mechanisms. In the same vein, Ahuja and Padhy (2021) show that ambiguity about what constitutes online sexual harassment remains a central barrier to both recognition and action.

Gómez-González et al. (2023) underscore a fundamental aspect: prevention and reporting tools should not be restricted solely to those directly affected, but must integrate the organizational climate and the disposition of bystanders, recognizing that informal support routes often precede formal reporting and may either facilitate or block it. Nonetheless, an effective strategy against digital gender-based violence requires transcending the strictly organizational plane through three fundamental axes: (1) education in critical digital skills and citizenship to mitigate training gaps and foster ethical use of platforms (Bastante Velázquez, 2026); (2) the deployment of macro-level public-awareness campaigns aimed at transforming democratic values in the digital arena and reversing the perception of impunity (Cuenca-Piqueras et al., 2020); and (3) the provision of specific digital self-protection training programs for women and girls that safeguard their privacy and ensure knowledge of their legal rights against cyberharassment (European Institute for Gender Equality [EIGE], 2017). This convergence of organizational intervention, proactive capacity building, and shared social responsibility is indispensable to mitigate the blurring of the spatiotemporal boundaries of the hybrid environment and to consolidate a safe and inclusive digital ecosystem.

### 4.5. Intervention: Survivor-Centered and Procedurally Credible Systems

It is essential to clarify that, in contrasting lifetime victimization with current preventive resources, the study does not aim to establish a direct causal link between the two variables. Rather, this analytical approach seeks to map the contemporary availability of organizational infrastructures against a population of women workers who, by their life trajectory, have historically been exposed to the continuum of gender-based violence. This contrast makes it possible to assess the current preventive-coverage gap in relation to the accumulated security needs of the working population. The finding that formal reporting to the police remains at only 1.7% signals an institutional failure that persists despite decades of legislative effort, reflecting low procedural credibility and high secondary-victimization costs that deter survivors from coming forward.

### 4.6. Strengths and Limitations

This study presents several strengths. It uses the most recent EU-GBV architecture available in the current working space, distinguishes analytically between in-person and online workplace harassment, and combines prevalence description with multilevel modeling. In addition, it incorporates dimensions of repetition, disclosure/reporting, and organizational preparedness that usually remain on the margins of strictly prevalence-based analyses.

Several limitations must also be made explicit. First, the analytical working file covers 17 countries, not the EU-27, so pan-European official figures were used as contextual references. Second, the measurement of online harassment depends on a limited number of items and probably underestimates emerging forms of digital victimization. Third, the multilevel associations do not identify causal mechanisms. Fourth, the transition to digital formats in surveys of this type may be associated with response-rate challenges for more sensitive questions, which calls for caution when conducting in-depth intersectional analyses of certain vulnerable groups. We rely on unweighted estimation to maximize the precision of the multilevel maximum-likelihood estimates; a design-weighted replication was beyond the present scope and is noted as a direction for future work. Age is treated as an analytical stratification (general versus under-35 models) rather than through multiplicative interaction terms, preserving parsimony and the interpretability of the reparameterized odds ratios; formal interaction testing (e.g., age × urbanization) is left to future modeling. The exploration of country-level macro predictors in extended models is likewise identified as a priority for future cross-national research.

The degree of urbanization is not available for Estonia in the EU-GBV: 70% of its subsample lacks this classification, and all cases without urbanization in the dataset correspond to this country. Consequently, the multilevel models adjusted for urbanization exclude these cases through listwise deletion, which reduces the analytical sample from 18,162 to 17,280 in the young cohort and from 84,950 to 81,760 in the general set, and decreases Estonia’s representation in the adjusted estimates and in the between-country variance. The urban-rural contrast is therefore estimated over the countries with available classification; cases without urbanization do not inform this contrast in any specification. Because the urbanization variable is structurally absent for most of the Estonian subsample rather than missing at the item level, multiple imputation is not applicable, and listwise deletion is the appropriate treatment.

A major limitation concerns the channel specification of the derived indicators. As documented in the EU-GBV methodological manual (Eurostat, 2021, KS-GQ-21-009), except for the items on staring and unsolicited physical contact, the wording of the items grouped in the in-person indicator does not specify whether the behavior occurred in person or through digital means. Their retention preserves the multi-item sensitivity of the construct, but it may misclassify technology-mediated occurrences; the most plausible consequence is an overestimation of the in-person component, while the online indicator—built exclusively from items explicitly naming a digital channel—is unaffected in its construction. The channel-ambiguous item on exposure to sexually explicit images or videos was assigned to neither indicator. A strict sensitivity analysis (Appendix A), re-estimating the in-person models with an indicator restricted to the two intrinsically face-to-face items (staring/leering and unsolicited physical contact), confirmed that the direction and magnitude of all effects remain unchanged; statistical significance is preserved for every covariate except the smallest migrant-origin contrasts, which retain the same direction with attenuated significance (in the general model, other-EU-born, *p* = 0.069, and foreign-born with country unknown, *p* = 0.059; in the young cohort, the already marginal foreign-born-with-country-unknown category, *p* = 0.154).

This channel ambiguity has a direct corollary for the hybrid indicator. Because hybrid victimization is operationalized as the co-occurrence of the in-person and online indicators, it inherits the channel ambiguity of its in-person component: episodes captured by channel-ambiguous items that were in fact technology-mediated would classify as hybrid a victimization that was strictly digital. The hybrid prevalences reported in Table 1 should therefore be read as upper bounds of strictly physical-plus-digital hybridization. Re-deriving the hybrid indicator under the strict definition of the in-person component (staring/leering and unsolicited physical contact only) quantifies this bound: the overall hybrid prevalence falls from 7.5% (6608/87,813) to 6.7% (5874/87,442 jointly valid cases), and the share of online-harassment victims who also report in-person victimization falls from 88.6% to 79.1%. The substantive reading is unaffected: even under the strict definition, four out of five women experiencing online harassment simultaneously report in-person victimization, so digital victimization continues to operate as a component of environmental continuity rather than as a substitute channel. The online component itself remains unaffected by construction, and the age-specific hybrid prevalences in Table 1, which were not re-derived, retain their upper-bound reading.

## 5. Conclusions

Workplace sexual harassment against women in Europe is best understood as a stratified, organizationally embedded, and context-sensitive phenomenon. In-person harassment remains the dominant substrate of victimization, but online forms and hybrid exposures show that digitalization expands—rather than replaces—prior configurations of power and risk. The greater concentration of online harassment among young women and the persistence of between-country variation support an interpretation that is simultaneously life-course-based and structural.

The central implication, therefore, is not simply that organizations should raise awareness, but that they must build integrated systems of prevention, education, and intervention that jointly address in-person and digital risk from a gender-sensitive perspective, a multivariable multilevel analysis attentive to intersecting axes (based on main effects and stratification of the young cohort), and a procedurally credible approach. Without training, designated contact persons, risk analysis, reliable disclosure/reporting routes, and sustained support, formal policy will remain more declarative than effective. Accordingly, public-awareness and educational campaigns should reject a one-size-fits-all model and be tailored to national context, emphasizing visibility and de-normalization in high-reporting Western clusters and strengthening digital-labour protections in Eastern European sectors facing high online risk.

## Figures and Tables

**Table 1:** Age gradient in lifetime sample prevalence of in-person, online, and hybrid workplace sexual harassment modalities.

Age Group	In-Person, % (*n/N*)	Online, % (*n/N*)	Hybrid, % (*n/N*)	Hybrid/Online %
18–24	48.2 (2863/5941)	19.0 (1125/5908)	17.4 (1029/5922)	91.5
25–34	44.3 (5728/12,916)	15.3 (1968/12,858)	13.8 (1781/12,880)	90.5
35–44	37.1 (5944/16,035)	10.6 (1687/15,978)	9.4 (1508/16,000)	89.4
45–54	31.9 (5805/18,190)	8.1 (1476/18,130)	7.2 (1302/18,162)	88.2
55–64	27.2 (5040/18,505)	4.6 (856/18,449)	3.9 (720/18,481)	84.1
65–74	19.8 (3238/16,392)	2.1 (346/16,344)	1.6 (268/16,368)	77.5
Total	32.5 (28,618/87,979)	8.5 (7458/87,667)	7.5 (6608/87,813)	88.6

Note: Denominators vary slightly across indicators owing to item-specific non-response. Source: authors’ own elaboration based on EU-GBV survey microdata (2021). The column ‘Hybrid/online %’ expresses the proportion of online-harassment victims who simultaneously experienced in-person victimization.

**Table 2:** Prevalence of workplace sexual harassment by country and modality: general population and young cohort (18–34 years).

Country	In-Person General (%)	In-Person <35 (%)	Online General (%)	Online <35 (%)
France (FR)	58.7	66.2	10.6	14.4
Finland (FI)	50.9	65.1	13.7	18.7
Slovakia (SK)	49.4	56.3	20.9	33.3
Denmark (DK)	43.3	56.6	9.0	15.0
Belgium (BE)	41.0	52.4	12.4	20.6
Greece (EL)	39.3	52.8	9.9	20.4
Croatia (HR)	34.7	46.0	12.5	24.7
Estonia (EE)	31.9	43.5	15.8	29.8
Slovenia (SI)	29.2	42.6	14.2	28.7
Austria (AT)	27.7	34.0	8.4	14.1
Spain (ES)	27.1	39.7	6.4	13.2
Malta (MT)	26.5	32.8	5.5	8.7
Lithuania (LT)	15.8	22.1	2.0	4.5
Poland (PL)	11.9	14.3	1.5	3.7
Portugal (PT)	11.0	19.9	1.9	3.8
Bulgaria (BG)	9.4	18.7	1.0	4.6
Latvia (LV)	9.2	14.5	1.2	2.1
Total (17 countries)	32.5	45.6	8.5	16.5

Note: Lifetime prevalence over the working life; proportions (mean of the dichotomous variable × 100). “General” = all ages (18–74 years); “<35” = women aged 18–34. Countries ordered by descending in-person general prevalence. Valid-response denominators vary slightly across indicators. Source: authors’ own elaboration based on EU-GBV survey microdata (2021).

**Table 3:** Individual correlates of in-person and online workplace sexual harassment in the general population of women (generalized linear mixed models).

Variable (Reference Category)	In-Person OR (95% CI)	Online OR (95% CI)
Age (ref.: 65–74 years)		
18–24 years	2.88 (2.68–3.09)	7.81 (6.85–8.91)
25–34 years	2.49 (2.35–2.64)	6.21 (5.49–7.02)
35–44 years	2.02 (1.91–2.14)	4.41 (3.90–4.99)
45–54 years	1.69 (1.60–1.78)	3.58 (3.17–4.06)
55–64 years	1.41 (1.34–1.49)	2.01 (1.76–2.29)
Educational level (ref.: High, ISCED 5–8)		
Low (ISCED 0–2)	0.58 (0.55–0.61)	0.73 (0.66–0.80)
Medium (ISCED 3–4)	0.78 (0.76–0.81)	0.92 (0.86–0.97)
Degree of urbanization (ref.: Rural)		
Cities	1.43 (1.38–1.49)	1.26 (1.18–1.35)
Towns and suburbs	1.17 (1.12–1.22)	1.16 (1.07–1.25)
Country of birth (ref.: Native-born)		
Another EU country	1.12 (1.03–1.22)	1.34 (1.19–1.52)
Outside the EU	0.81 (0.76–0.87)	0.98 (0.88–1.11) n.s.
Financial strain (ref.: Can cope with an unexpected expense)		
Cannot cope with the expense	1.07 (1.03–1.12)	1.30 (1.23–1.39)
σ^2^ country (SE)	0.637 (0.226); *p* = 0.005	0.972 (0.348); *p* = 0.005
Adjusted ICC	12.3%	6.8%
R^2^ (marginal/conditional)	0.039/0.157	0.035/0.101
*N*	81,760	81,531

Note: Generalized linear mixed models (binomial distribution, logit link) with a random intercept by country (17 countries). Odds ratios (OR) and 95% confidence intervals are shown for the probability of having experienced harassment, P(Y = 1). All ORs are significant (*p* < 0.05; the 95% CI excludes 1.00) except those marked “n.s.” σ^2^ country = variance of the random intercept by country (SE = standard error); ICC = intraclass correlation coefficient (computed using the delta method); R^2^ = Nakagawa’s pseudo-R^2^. Source: authors’ own elaboration based on EU-GBV survey microdata (2021). ISCED = International Standard Classification of Education.

**Table 4:** Correlates of in-person and online workplace sexual harassment in the cohort of young women (18–34 years) (generalized linear mixed models).

Variable (Reference Category)	In-Person OR (95% CI)	Online OR (95% CI)
Age (ref.: 25–34 years)		
18–24 years	1.10 (1.02–1.18)	1.22 (1.11–1.34)
Educational level (ref.: High, ISCED 5–8)		
Low (ISCED 0–2)	0.72 (0.64–0.82)	0.94 (0.80–1.12) n.s.
Medium (ISCED 3–4)	0.91 (0.85–0.98)	1.14 (1.03–1.25)
Degree of urbanization (ref.: Rural)		
Cities	1.49 (1.37–1.63)	1.29 (1.14–1.45)
Towns and suburbs	1.15 (1.04–1.26)	1.11 (0.98–1.26) n.s.
Country of birth (ref.: Native-born)		
Another EU country	1.20 (1.01–1.42)	1.39 (1.13–1.71)
Outside the EU	0.84 (0.73–0.97)	1.16 (0.96–1.40) n.s.
Financial strain (ref.: Can cope with an unexpected expense)		
Cannot cope with the expense	1.00 (0.92–1.08) n.s.	1.12 (1.01–1.23)
σ^2^ country (SE)	0.658 (0.236); *p* = 0.005	0.924 (0.339); *p* = 0.006
Adjusted ICC	14.0%	11.0%
R^2^ (marginal/conditional)	0.010/0.149	0.004/0.114
*N*	17,280	17,212

Note: Specification identical to Table 3, restricted to women aged 18–34. OR and 95% CI for P(Y = 1) with a random intercept by country (17 countries). “n.s.” = not significant (the 95% CI includes 1.00). σ^2^ country, ICC, and R^2^ defined as in Table 3. The mode of data collection is excluded because it is confounded with the country level. Source: authors’ own elaboration based on EU-GBV survey microdata (2021).

**Table 5:** Indicators of repetition, disclosure/reporting, and organizational preparedness in relation to sexual harassment experiences.

Dimension	Indicator	*n*/Denominator	%
Repetition	Repeated sexual harassment by the same perpetrator	15,742/27,223	57.8
Recency	Most recent episode within the last 12 months	4084/28,489	14.3
Informal disclosure	Talked to someone close	2428/4084	59.5
Semi-formal disclosure	Talked to a coworker	2026/4084	49.6
Workplace disclosure	Talked to a manager or supervisor	944/4084	23.1
Formal report	Reported to the police	71/4084	1.7
Formal report	Reported to another official body	48/4084	1.2
Prevention	Training available at work	11,241/47,981	23.4
Prevention	Designated contact person available	17,839/47,981	37.2
Help-seeking knowledge	Knows where to seek help	56,697/85,058	66.7

Note: Denominators vary by indicator: repetition and recency are computed over the valid responses for each item (*n* = 27,223 and *n* = 28,489, respectively); disclosure and reporting, over the women who experienced harassment in the previous 12 months (*n* = 4084); training and contact person, over the subsample to which the workplace-prevention module was administered (*n* = 47,981); and knowledge of where to seek help, over valid responses (*n* = 85,058). Source: authors’ own elaboration based on EU-GBV survey microdata (2021).

## Data Availability

The data used in this study come from the EU-GBV survey microdata. Their availability is subject to the access conditions established by the data provider(s).

## Appendix A. Sensitivity Analysis of the In-Person Indicator

The sensitivity analysis was extended to its strictest form: the in-person models were re-estimated using an indicator restricted to the only two items whose official wording is intrinsically face-to-face, namely inappropriate staring or leering and unsolicited physical contact, thereby excluding the four channel-ambiguous items (indecent sexual jokes or offensive remarks; inappropriate suggestions to go out on a date; inappropriate suggestions for any sexual activity; and threats of unpleasant consequences for refusing sexual proposals or advances). The prevalence of the strict indicator is 27.7% (24,298/87,728), compared with 32.5% (28,618/87,979) for the full six-item indicator, and it captures 85.1% of the women flagged by the full indicator among jointly valid cases. Models replicate the specification of Table 3 and Table 4 (binomial logistic generalized linear mixed models with a random intercept for country; cases with an unclassified degree of urbanization excluded) and were estimated with the same procedure used for Table 3 and Table 4 (GENLINMIXED, IBM SPSS); the original models were re-estimated in the same run to ensure strict comparability, so point estimates may differ marginally from Table 3 and Table 4. Estimation samples: *N* = 81,919 (original) and *N* = 81,731 (strict) in the general population; *N* = 17,308 and *N* = 17,271 in the young cohort. The between-country variance is essentially unchanged (0.637 vs. 0.625 in the general model; 0.658 vs. 0.609 in the young model). Table A1 and Table A2 report the fixed effects. The direction and magnitude of all effects remain unchanged under the strict indicator. In the general-population model, statistical significance is preserved for every covariate except the two smallest migrant-origin contrasts, which attenuate to marginal significance while retaining the same direction (other EU country: OR 1.09, *p* = 0.069; foreign-born with country unknown: OR 0.66, *p* = 0.059). In the young-cohort model, every contrast that was significant in the original specification remains significant under the strict indicator, including the non-EU-born category (OR 0.86, *p* = 0.047); the small foreign-born-with-country-unknown category, already marginal in the original specification (*p* = 0.059), attenuates further (*p* = 0.154), and the economic-strain coefficient remains null in both specifications (OR 1.00 vs. 1.05; *p* > 0.2). An intermediate specification excluding only the indecent-jokes item, reported in the previous round of revision, yielded the same conclusions.

**Table A1:** General population: original vs. strict in-person indicator (odds ratios and 95% confidence intervals).

Predictor	Original OR (95% CI)	*p*	Sensitivity OR (95% CI)	*p*
Intercept	0.53 (0.36–0.77)	0.001	0.40 (0.27–0.58)	<0.001
Age 25–34	0.87 (0.81–0.93)	<0.001	0.87 (0.81–0.94)	<0.001
Age 35–44	0.70 (0.66–0.75)	<0.001	0.72 (0.67–0.77)	<0.001
Age 45–54	0.59 (0.55–0.63)	<0.001	0.61 (0.57–0.66)	<0.001
Age 55–64	0.49 (0.46–0.53)	<0.001	0.53 (0.49–0.57)	<0.001
Age 65–74	0.35 (0.32–0.37)	<0.001	0.37 (0.34–0.40)	<0.001
Education: Medium (ISCED 3–4)	1.35 (1.28–1.42)	<0.001	1.34 (1.27–1.42)	<0.001
Education: High (ISCED 5–8)	1.73 (1.63–1.82)	<0.001	1.64 (1.55–1.74)	<0.001
Urbanization: Town and suburbs	0.81 (0.78–0.85)	<0.001	0.83 (0.79–0.86)	<0.001
Urbanization: Rural areas	0.70 (0.67–0.73)	<0.001	0.71 (0.68–0.74)	<0.001
Country of birth: Other EU country	1.12 (1.03–1.22)	0.010	1.09 (0.99–1.19)	0.069
Country of birth: Non-EU country	0.81 (0.76–0.87)	<0.001	0.82 (0.76–0.88)	<0.001
Country of birth: Foreign-born but country unknown	0.65 (0.43–0.97)	0.033	0.66 (0.42–1.02)	0.059
Unable to afford unexpected expenses	1.07 (1.03–1.12)	<0.001	1.09 (1.04–1.13)	<0.001

**Table A2:** Young cohort (18–34): original vs. strict in-person indicator (odds ratios and 95% confidence intervals).

Predictor	Original OR (95% CI)	*p*	Sensitivity OR (95% CI)	*p*
Intercept	0.62 (0.41–0.94)	0.023	0.47 (0.32–0.70)	<0.001
Age 25–34	0.91 (0.84–0.98)	0.010	0.91 (0.84–0.98)	0.011
Education: Medium (ISCED 3–4)	1.27 (1.12–1.43)	<0.001	1.22 (1.08–1.39)	0.002
Education: High (ISCED 5–8)	1.39 (1.23–1.58)	<0.001	1.31 (1.15–1.49)	<0.001
Urbanization: Town and suburbs	0.77 (0.71–0.84)	<0.001	0.81 (0.74–0.88)	<0.001
Urbanization: Rural areas	0.67 (0.61–0.73)	<0.001	0.69 (0.64–0.76)	<0.001
Country of birth: Other EU country	1.19 (1.01–1.42)	0.042	1.24 (1.04–1.47)	0.014
Country of birth: Non-EU country	0.84 (0.73–0.97)	0.020	0.86 (0.75–1.00)	0.047
Country of birth: Foreign-born but country unknown	0.44 (0.19–1.03)	0.059	0.53 (0.22–1.27)	0.154
Unable to afford unexpected expenses	1.00 (0.92–1.08)	0.926	1.05 (0.97–1.13)	0.221

Note. Reference categories: age 18–24; low education (ISCED 0–2); cities; born in reporting country; able to afford unexpected expenses.
